# Repeat Ablation for Persistent Atrial Fibrillation: CFA/DOA-Guided Substrate Ablation Versus Re-PVI Alone in the Prematurely Terminated Randomized AF-CAM Trial

**DOI:** 10.3390/jcm15208010

**Published:** 2026-10-15

**Authors:** Stephan Willems, Pawel Kuklik, Peter Wohlmuth, Melanie A. Gunawardene, Jens Hartmann, Jannis Dickow, Tim Harloff, Kathrin Heitmann, Christian Meyer, Katharina Scherschel, Susanne Scholz, Arian Sultan, Rahin Wahedi, Arso Zahir-Akkra, Nele Gessler

**Affiliations:** 1 Department of Cardiology and Intensive Care Medicine, Faculty of Medicine, Semmelweis University Campus Hamburg, Asklepios Hospital St. Georg, 20099 Hamburg, Germany; 2 Atrial Fibrillation Network (AFNET), 48149 Münster, Germany; 3 Asklepios Proresearch, Research Institute, 20099 Hamburg, Germany; 4 Evangelical Hospital Duesseldorf, 40217 Duesseldorf, Germany; 5 Heart Center, Cologne University Hospital, 50937 Cologne, Germany; 6 Medical School Hamburg, 20457 Hamburg, Germany

**Keywords:** atrial fibrillation, catheter ablation, randomized study

## Abstract

**Background and Aims:** Currently, no standard approach exists for substrate ablation in persistent atrial fibrillation (AF). This study aimed to assess whether repeat pulmonary vein isolation (Re-PVI) plus targeted substrate ablation improves long-term outcomes compared with Re-PVI alone in patients with persistent AF undergoing a second ablation. **Methods:** The AF-CAM study is an investigator-initiated, prospective, randomized, open-label, blinded-endpoint, multi-center clinical trial. Patients with persistent AF undergoing a second ablation for arrhythmia recurrence were randomized 1:1 to either Group A (Re-PVI alone) or Group B (Re-PVI plus targeted ablation). The primary endpoint was freedom from any atrial arrhythmia (>30 s) between 3 and 12 months of follow-up. **Results:** Between August 2021 and November 2023, 56 patients (mean age 68 ± 10 years, 32% women) were randomized before the trial was terminated prematurely. There was no evidence of a difference in arrhythmia recurrence between the treatment groups (hazard ratio [HR] for recurrence, Group B vs. Group A: 1.07; 95% confidence interval [CI] 0.50 to 2.33; *p* = 0.85). Pulmonary vein reconnection was present in 86% of patients and was not associated with the risk of atrial arrhythmia recurrence (HR 0.927, 95% CI [0.275, 3.120]). Across both groups, catheter ablation was associated with improvements in quality of life (AFEQT score) and reductions in BNP levels during follow-up. **Conclusions:** In this prematurely terminated randomized trial, adding CFA- and DOA-guided substrate ablation to Re-PVI did not demonstrate an improvement in freedom from atrial arrhythmia compared with Re-PVI alone. The small sample size and imprecise treatment estimate preclude a definitive conclusion about the efficacy of the adjunctive strategy. AFEQT scores increased and BNP levels decreased over follow-up in the studied patients. Larger studies are needed to further evaluate the complex atrial substrate in persistent AF.

## 1. Introduction

Atrial fibrillation (AF) is the most common cardiac arrhythmia, imposing a significant burden from associated morbidity, mortality, and healthcare costs [1]. While catheter ablation, primarily pulmonary vein isolation (PVI), is a cornerstone therapy for paroxysmal AF, durable rhythm control in persistent AF remains a challenge [2,3]. The progression from paroxysmal AF to persistent AF involves significant atrial remodeling, creating a complex substrate that sustains the arrhythmia independently of pulmonary vein triggers [4].

Consequently, the optimal ablation strategy for persistent AF, particularly after initial PVI failure, is actively debated [5,6]. While Re-PVI remains essential, this approach alone often yields suboptimal long-term success [7]. Various adjunctive substrate strategies have been proposed, targeting phenomena like complex fractionated atrial electrograms (CFAE), low-voltage zones, rotors, or autonomic ganglia [8,9,10,11,12,13,14,15]. However, large trials like STAR AF II failed to show a clear benefit of empiric CFAE or linear ablation added to PVI [2,4], leaving uncertainty about the value of substrate ablation. Recent trials like ERASE-AF [16] and PRECEPT [17] also showed heterogeneous success compared to other approaches. This uncertainty necessitates refined strategies targeting specific, mechanistically plausible substrate components [18].

Recent high-resolution mapping systems allow detailed characterization of the atrial substrate. Our group and others have explored mapping to identify complex, continuous electrical activity hypothesized as drivers of persistent AF [15,19]. The AF-CAM study hypothesized that targeting specific electrogram characteristics—namely, continuous fractionated activity (CFA) and high spatiotemporal dispersion of activity (DOA) with a high-density mapping system—added to Re-PVI improves outcomes in repeat persistent AF ablation. These parameters were chosen to capture diverse AF drivers, including micro-reentry, fibrillary conduction, or pivoting waves.

CFA quantifies continuous fractionated activity via a sliding-window algorithm, potentially identifying fibrillatory conduction or micro-reentry; it is distinct from traditional CFAE metrics [9], focusing on Min/Max ratios in range-transformed signals. DOA is conceptually related to, but methodologically distinct from, the spatiotemporal electrogram dispersion described by Seitz et al. [15]. Whereas the latter is identified visually as clusters of adjacent bipoles with activation spanning the AF cycle length, DOA is an automatically calculated continuous index (0–1) quantifying the fraction of recording time occupied by local electrical activity across a group of simultaneously recorded bipolar electrograms. These metrics were selected over empiric CFAE [2] based on pilot data suggesting capture of mechanistically plausible drivers [15,19].

Therefore, the AF-CAM trial is a prospective, randomized, multicenter study comparing Re-PVI alone versus Re-PVI combined with targeted CFA and DOA ablation in persistent AF patients undergoing a second ablation. We hypothesized this adjunctive substrate ablation would improve 12-month freedom from atrial arrhythmias compared to Re-PVI alone.

## 2. Methods

### 2.1. Study Design and Oversight

We conducted an investigator-sponsored, prospective, randomized, open-label, blinded-endpoint, multicenter clinical trial in Germany (NCT05496088). The trial was opened for recruitment at three centers in Germany; two centers enrolled patients. The study complied with the Declaration of Helsinki and Good Clinical Practice and the protocol was approved by each participating center’s ethics committee. A steering committee oversaw the trial. The study was supported by a research grant for investigator-sponsored research from Boston Scientific, which had no role in study design, data analysis, or manuscript preparation. No independent data monitoring committee was involved in the decision to stop recruitment. The funder discontinued financial support because of slow recruitment. The steering committee was involved in the subsequent termination process. At the time of the decision, no party had access to treatment-group-specific outcome data. No independent data monitoring committee was involved.

Endpoint adjudication was performed by investigators blinded to treatment allocation. The authors attest to the accuracy of the data and analyses and to the fidelity of this report to the trial protocol; all data that support the study findings are available on request.

### 2.2. Patient Population

Eligible patients were adults (18–85 years) with documented persistent AF, defined as an episode sustained >7 days but <1 year, or <7 days requiring cardioversion after >48 h. All participants had one previous PVI-only ablation procedure at least 3 months, but not more than 10 years, prior to enrolment.

Patients were excluded from the study if they presented with atrial fibrillation (AF) due to reversible causes; continuous AF lasting longer than 12 months; or AF secondary to electrolyte imbalance, thyroid disease, or other reversible or non-cardiac causes. Cardiovascular contraindications included any cardiac surgery within the past 2 months (60 days); documented left atrial (LA) thrombus on imaging; LA size greater than 60 mm; myocardial infarction within the past 2 months (60 days); a documented thromboembolic event (including TIA) within the past 12 months; rheumatic heart disease; uncontrolled heart failure or NYHA function class III or IV; unstable angina; or if the patient was awaiting cardiac transplantation or other cardiac surgery within the next 12 months (365 days). Broader exclusion criteria encompassed any contraindication to anticoagulation (heparin or warfarin); acute illness or active systemic infection or sepsis; diagnosed atrial myxoma; significant severe pulmonary disease with chronic symptoms; and patients with hemodialysis. Additionally, women who were pregnant, patients with a significant congenital anomaly or medical problem that would preclude enrolment, and individuals with an intramural thrombus, tumor, or other abnormality or condition that precludes vascular access or catheter manipulation were also excluded.

### 2.3. Enrolment and Randomization

Written informed consent was obtained before the procedure. Re-PVI was performed before randomization. Immediately after Re-PVI, rhythm status and AF inducibility were assessed to confirm eligibility for randomization. According to the protocol, only patients with ongoing or inducible AF were randomized. For these patients, treatment allocation was generated and revealed during the procedure using a computer-based randomization tool with variable block sizes, stratified by center. Patients in sinus rhythm with non-inducible AF were to be classified as dropouts before randomization.

### 2.4. Ablation Procedure

Mapping was performed using a high-resolution 8-spline multipolar basket catheter after a pre-procedural TEE excluded LA thrombus (RHYTHMIA mapping system; Boston Scientific Corporation, Marlborough, MA, USA). RF ablation was delivered via an irrigated-tip catheter at 25–35 W (≤25 W posteriorly). Procedures were performed at experienced centers following ESC anticoagulation guidelines [1].

In both groups, any reconnected pulmonary veins were assessed using a circular mapping catheter and re-isolated via circumferential ablation to achieve entrance block.

Group A (Re-PVI Only): After Re-PVI, no further ablation was performed; if AF persisted, electrical cardioversion was used.
Group B (Re-PVI + Substrate Ablation): If AF remained ongoing or inducible post-Re-PVI, a high-density substrate map of both atria was created using the Orion™ 64-electrode basket catheter (Boston Scientific Corporation, Marlborough, MA, USA).

### 2.5. Electrical Substrate Characterization and Analysis

**Figure 1:**
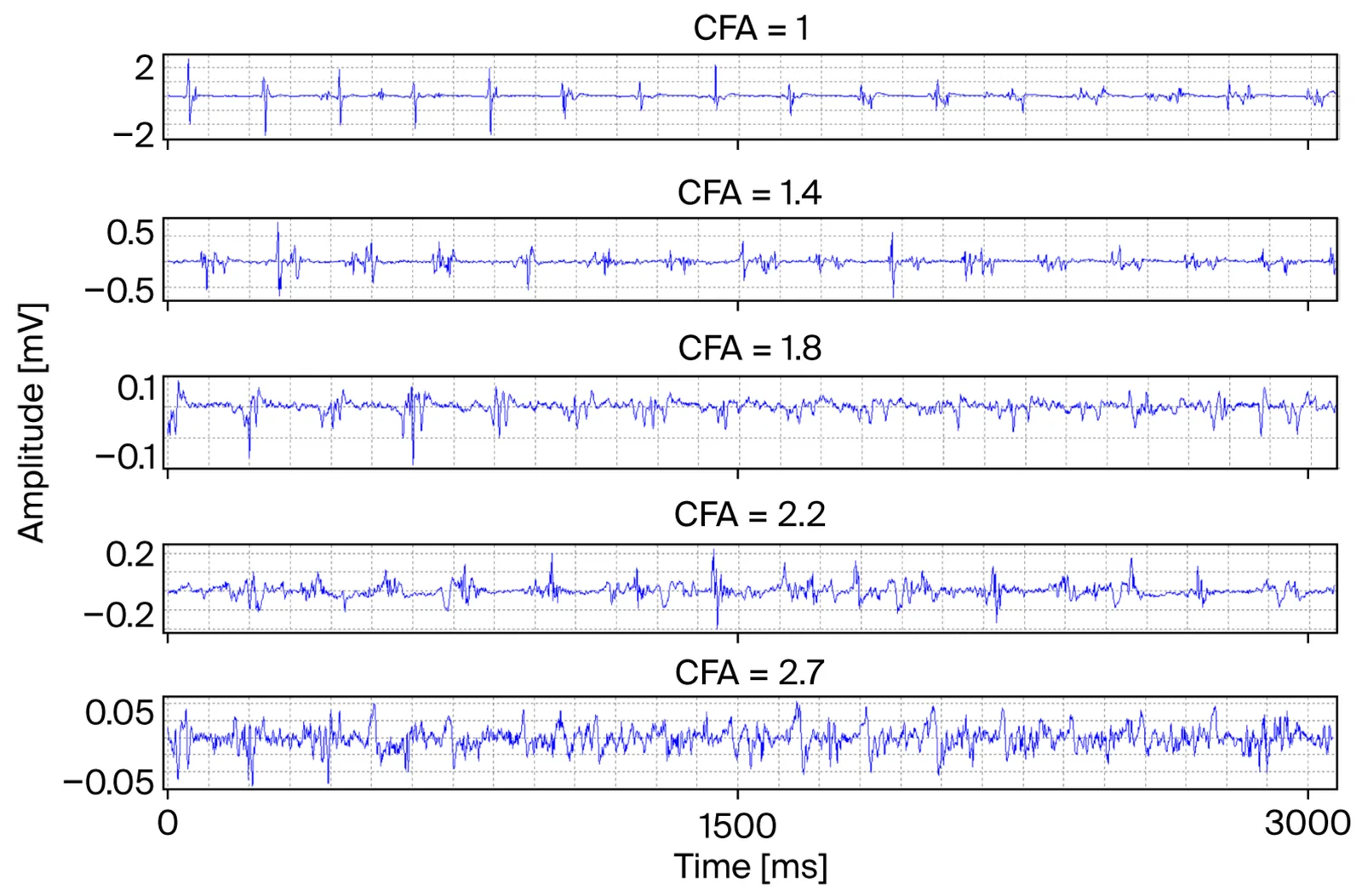
Example bipolar atrial electrograms with varying degrees of continuous fractionation and corresponding continuous fractionated activity (CFA) values. Examples are derived from 5-s recordings acquired at 1 kHz using standard Rhythmia-recommended bipolar acquisition filtering, with no additional offline filtering. CFA was calculated from the mean Min/Max ratio of the 50-ms range-transformed signal within consecutive 500-ms segments; higher values indicate more pronounced continuous fractionated activity.

Custom algorithms were used to calculate two parameters for each acquired EGM point:

Continuous fractionated activity (CFA): Quantified the degree of continuous fractionated EGM morphology, irrespective of amplitude, potentially identifying areas of disorganised conduction such as fibrillatory conduction or micro-reentry (see Figure 1).

**Figure 2:**
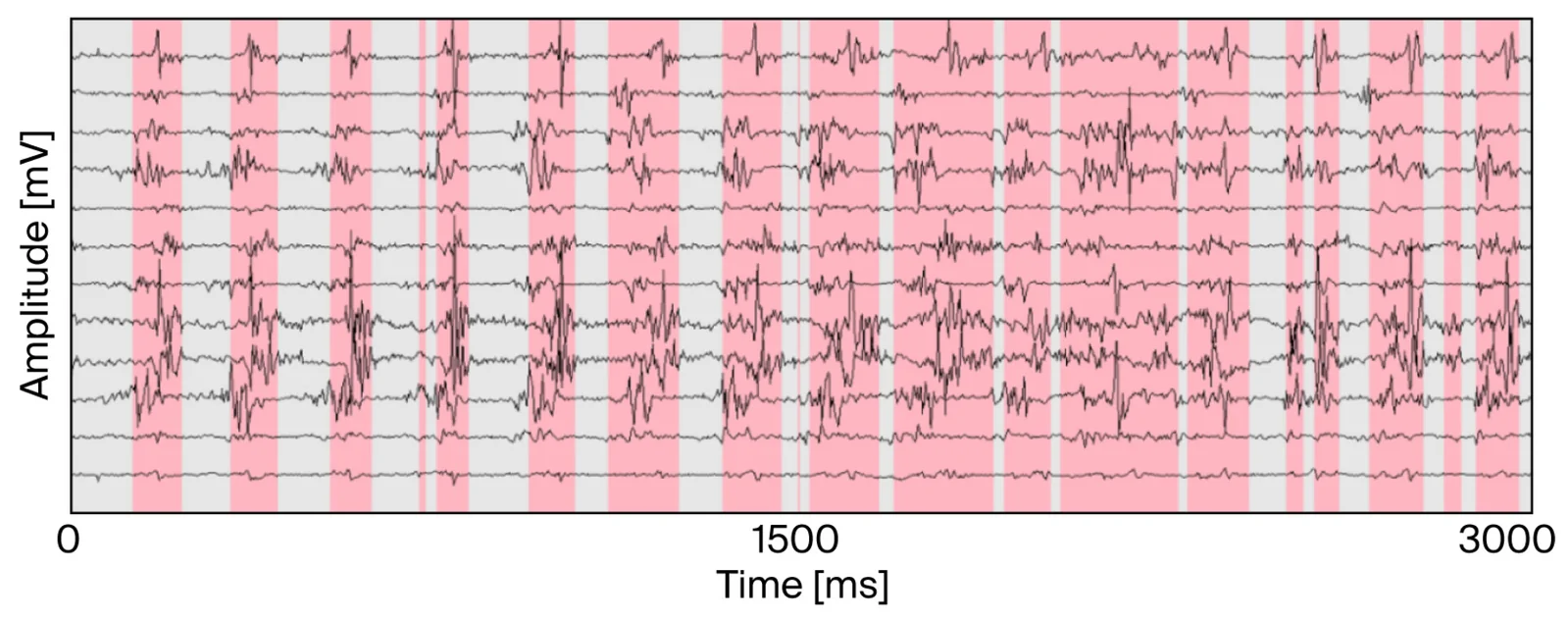
Estimation of dispersion of activity (DOA) from a representative group of simultaneously recorded bipolar atrial fibrillation electrograms. Recordings were 5 s in duration, sampled at 1 kHz, and acquired using standard Rhythmia-recommended bipolar acquisition filtering. Red intervals denote time classified as electrically active around detected local activations; gray intervals denote periods during which all valid electrograms in the group were silent. The activity half-width was set dynamically to AFCL/(2N), where AFCL is the mean atrial fibrillation cycle length and N is the number of valid bipolar electrograms in the group. DOA = 1 − silent-time fraction and ranges from 0 to 1, with higher values indicating greater temporal continuity of spatially distributed electrical activity.

Dispersion of activity (DOA): An automatically calculated continuous index quantifying the fraction of recording time occupied by local electrical activity across simultaneously recorded valid bipolar electrograms, potentially identifying regions with persistent spatially distributed activity (see Figure 2).

Electrogram acquisition and preprocessing: Bipolar electrograms were formed from electrode pairs along individual Orion splines (Boston Scientific Corporation, Marlborough, MA, USA). Five-second recordings were acquired at each recording location, processed at a sampling frequency of 1 kHz, and the complete 5-s recording was used for analysis. Standard Rhythmia-recommended bipolar acquisition filtering was used, with no additional offline filtering. Before CFA and DOA calculation, an electrogram was retained only if its absolute amplitude exceeded 0.05 mV at least once in every consecutive 500-ms segment of the recording; electrograms not meeting this criterion were excluded. No dedicated ventricular far-field subtraction or ventricular blanking was applied.

Map acquisition and quality: The operating electrophysiologist was instructed to cover the entire mappable surface of each atrium, aiming for approximately two to three recordings at each anatomical location. Because mapping was performed intraprocedurally, no prospectively specified numerical minimum mapping density or global quantitative map-quality threshold was imposed; map coverage and acceptability were assessed by the operating electrophysiologist.

Both parameters were quantified from local bipolar electrograms and interpolated onto the surface for visualization. The CFA parameter was computed using a sliding-window algorithm to quantify local signal fragmentation. First, a range-transformed signal was generated by calculating the local max-min difference within a moving 50 ms window, capturing rapid amplitude fluctuations. This range signal was then divided into 500 ms segments. For each segment, the ratio of the minimum to maximum range (Min/Max) served as a local fractionation measure. The final CFA index was the average of all Min/Max ratios, quantifying fine-scale amplitude variability; higher values indicated more pronounced continuous fractionated activity. Local activation detection and AF cycle-length estimation: Local activations were identified using a bipolar adaptation of sinusoidal recomposition. For each bipolar electrogram, a sinusoid with a nominal period of 180 ms was weighted by the absolute electrogram amplitude and contributions were summed across the recording to generate a recomposed signal. The Hilbert phase of the recomposed signal was calculated using a 256-ms convolution kernel, and local deflections were identified from negative phase transitions using a relative threshold of 10% of the local maximum evaluated over an adaptive 500-ms window, with an 80-ms blanking period. AF cycle length (AFCL) was then calculated as the mean interval between consecutive detected local activations.

Dispersion of activity (DOA) was defined as 1 minus the fraction of ‘silent’ time within each recording group (see Figure 2). Each group comprised the valid bipolar electrograms acquired simultaneously at a given recording location. Time around every detected local activation was labelled ‘active’ using a symmetric blanking half-width of AFCL/2N, where AFCL was the mean cycle length estimated from detected local activations and N was the number of valid bipolar electrograms in the group. The union of active periods across all electrograms in the group was used to determine the silent-time fraction. DOA (range 0–1) was calculated as 1 − (silent-time fraction). Recording groups with fewer than eight valid electrograms after signal-quality exclusion were not included in DOA analysis.

Spatial interpolation: CFA and DOA values at measured locations were propagated over the atrial surface mesh using an iterative diffusion-based, neighbour-averaging interpolation scheme. Values at nodes corresponding to measured data were kept fixed, whereas each unmeasured node was iteratively updated to the arithmetic mean of valid directly connected neighbouring node values. Iteration continued until the relative update threshold of 0.005 was no longer exceeded. This corresponds to a discrete Laplacian/harmonic interpolation of the measured scalar values over the surface mesh with measured values held fixed.

**Figure 3:**
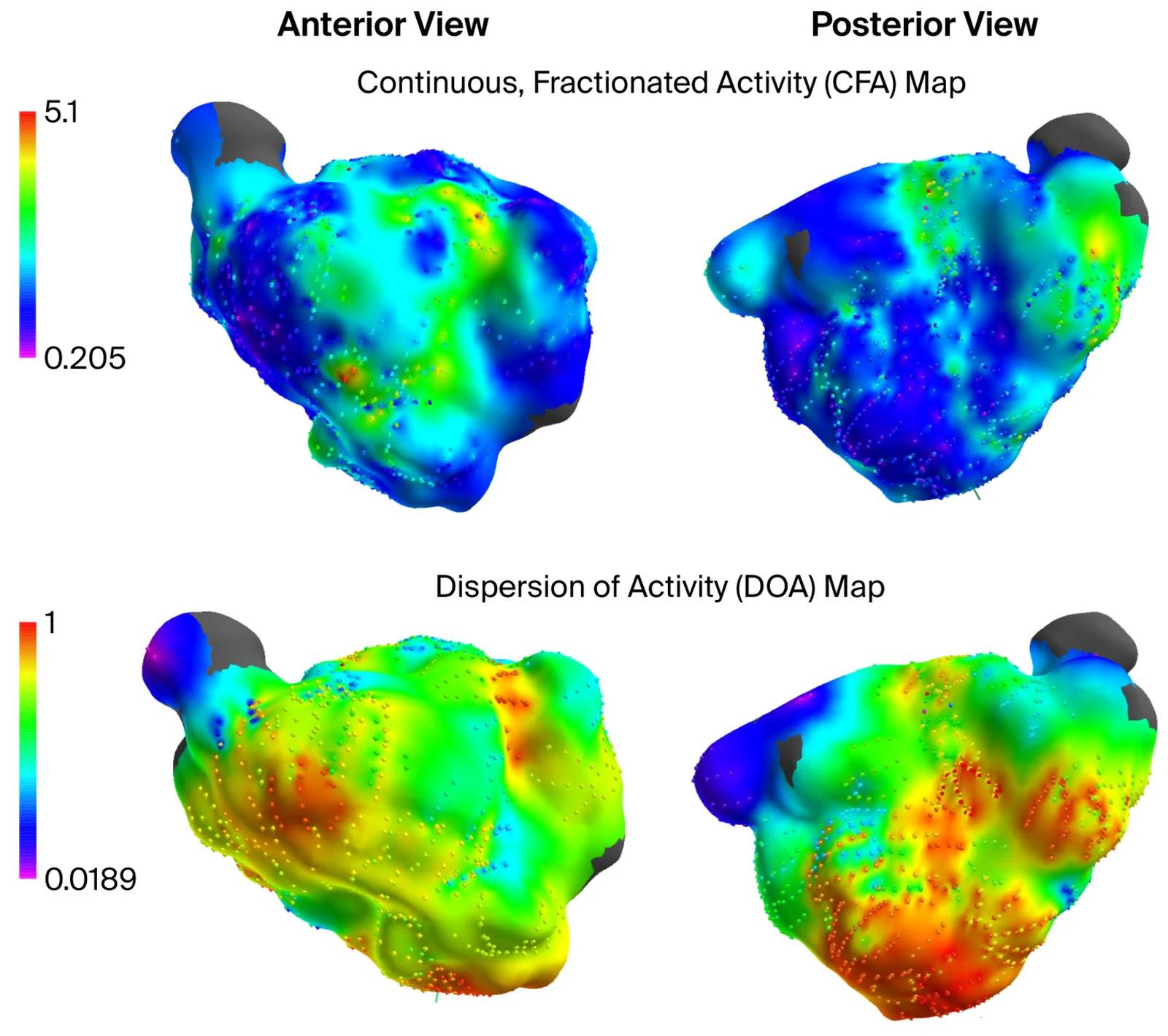
Representative anterior and posterior views of interpolated continuous fractionated activity (CFA; **left**) and dispersion of activity (DOA; **right**) maps. Maps are color-coded from lower values (purple/blue) to higher values (yellow/red); the numerical lower and upper limits of the displayed range are shown adjacent to each color bar. The underlying electrograms were acquired as 5-s recordings at 1 kHz using standard Rhythmia-recommended bipolar acquisition filtering. Ablation targets are not separately overlaid in this illustrative figure. During the procedure, target regions were defined independently within each atrium as the highest 10% of CFA values and the highest 10% of DOA values; regions meeting either criterion were targeted, and spatial overlap was not required.

Spatial CFA and DOA maps were generated (Figure 3).

Within each atrium, target regions were defined independently as regions containing the highest 10% of CFA values and regions containing the highest 10% of DOA values. Spatial overlap between CFA and DOA target regions was not required; regions meeting either criterion were targeted. These regions underwent RF ablation until local EGM amplitude was <0.1 mV. If organized atrial tachycardia (AT) occurred, it was mapped and ablated. External cardioversion was used if sinus rhythm was not achieved via ablation. PVI was re-confirmed before concluding the procedure.

All 28 patients randomized to Group B underwent bilateral atrial CFA/DOA mapping and subsequent substrate ablation according to the prespecified mapping criteria. CFA and/or DOA target regions were identified in all patients. Because target regions were defined using percentile-based criteria derived from continuous CFA and DOA maps, target presence was not treated as a separate procedural endpoint. Quantitative measures such as the number or surface area of individual target regions and total substrate lesion burden were not prospectively collected.

### 2.6. Follow-Up

After the index procedure, patients entered a 3-month blanking period, after which anti-arrhythmic drug therapy was discontinued per standard practice. During blanking period, the use of antiarrhythmic drug therapy, cardioversion, and medication was at the discretion of the investigator and according to current guidelines. Follow-up occurred at 3, 6, and 12 months. Each visit included collection of arrhythmia symptoms, cardiovascular medications, adverse events, and vital signs, plus a 12-lead ECG and 48-h Holter monitoring. Transthoracic echocardiography (TTE) assessing LA size and LVEF, blood tests (BNP), and the AFEQT questionnaire were performed at baseline and 12 months; TTE and blood tests were repeated at 6 months. Unscheduled visits were conducted for symptomatic episodes to document arrhythmia recurrence.

### 2.7. Endpoints

The primary efficacy endpoint was freedom from any documented atrial arrhythmia (AF, atrial flutter, or atrial tachycardia) lasting >30 s, assessed between 3 and 12 months post-ablation via 12-lead ECG, 48-h Holter monitoring, or symptom-driven event monitoring. At each site, one investigator blinded to treatment allocation reviewed the original ECG and Holter recordings.

Secondary efficacy endpoints included changes in LVEF and LA size by TTE, change in BNP levels, quality of life (AFEQT score), and medication use (AAD, anticoagulants), plus AF-related hospitalizations and clinical adverse events. Safety endpoints included serious adverse events. Ancillary endpoints (Group B only) included AF voltage, continuous fractionated activity (CFA), AF cycle length, and dispersion of activity (DOA).

### 2.8. Statistical Analysis

The sample size calculation was based on the log-rank test for comparison of time to atrial arrhythmia recurrence between treatment groups. Assuming freedom from atrial arrhythmia recurrence of 40% in the control group and 60% in the intervention group, a two-sided significance level of 0.05, 80% power, and 1:1 treatment allocation, 188 patients were required. To account for an anticipated dropout rate of 6%, the target sample size was increased to 200 patients. Continuous data are presented as median [Q1–Q3] for non-normally distributed data or mean ± SD for approximately normally distributed data. Categorical variables are reported with percentages (n). Procedural data were compared between groups. For continuous data, Wilcoxon rank-sum tests were performed, and for categorical variables, chi-square tests or Fisher’s exact test, when expected cell counts are lower than five, were performed.

The primary efficacy analysis was performed in accordance with the intention-to-treat principle and included all randomized patients analyzed according to the treatment group to which they were randomized. The primary endpoint was defined as the time to atrial arrhythmia recurrence, where the first qualifying atrial arrhythmia event per patient was considered. The primary endpoint was analyzed using a Cox proportional hazards model, with treatment group as a fixed effect and study center as a stratification factor. The treatment effect was summarized by the hazard ratio (HR) with corresponding 95% confidence interval (CI) and a Wald test *p*-value. Furthermore, a per-protocol analysis, excluding six patients not meeting the study inclusion criteria, was performed. To evaluate the association between pulmonary vein reconnection and arrhythmia recurrence, the Cox model for the ITT analysis was extended with the pulmonary vein reconnection status as a covariate. The reverse Kaplan–Meier method was used to estimate median follow-up time. Kaplan–Meier curves for the treatment groups were shown, representing freedom from atrial arrhythmia recurrence.

The primary efficacy outcome was analysed separately. Additional analyses were classified as prespecified or post hoc according to the trial protocol and statistical analysis plan.

The secondary outcomes, LVEF, left atrial diameter, and BNP, were analysed using separate linear mixed-effects models including treatment group, follow-up visit (6 or 12 months), the treatment-by-visit interaction, and the baseline value of the respective outcome as fixed effects. Study site and patient were included as random intercepts. Because AFEQT was assessed at only one post-baseline visit, its 12-month value was analysed using a baseline-adjusted model without a visit term, treatment-by-visit interaction, or patient-level random intercept. BNP was log-transformed before analysis; adjusted between-group contrasts were exponentiated and reported as ratios of Group B to Group A. For each outcome and visit, adjusted between-group estimates are reported with 95% confidence intervals and *p*-values.

Furthermore, the differences in secondary endpoints at the follow-up visits, compared to baseline values, were analysed within each treatment group. The data were processed according to the MMRM (Mixed Model for Repeated Measures) style, where the baseline is treated as another observation time point. The linear mixed-effects models included a treatment-by-visit interaction and patient-level and study site as random intercepts. Least-squares mean changes from baseline within each treatment group were derived from the fitted MMRM and are presented with corresponding 95% confidence intervals and nominal *p*-values. Missing outcome data were not imputed. Linear mixed-effects models were fitted using maximum likelihood estimation, which incorporates all available observations under the assumption that data were missing at random (MAR).

Regional mapping parameters were summarized descriptively for Group B. No formal statistical comparisons between atrial regions were performed. The numbers of patients and evaluable maps contributing to each chamber- and region-specific summary are reported separately.

Safety analyses included all reported serious adverse events (SAEs). SAEs were summarized descriptively by treatment group as counts and percentages of patients with at least one event. No adjustment for multiplicity was applied to safety analyses.

Multiplicity adjustments were not applied. Formal statistical testing for secondary endpoints was performed at a two-sided significance level of alpha = 0.05 without multiplicity adjustment. Consequently, the reported *p*-values and corresponding 95% confidence intervals for all secondary endpoints are of a descriptive and exploratory nature. Any interpretations derived from these secondary analyses should be considered supportive and hypothesis-generating rather than definitive or confirmatory.

All statistical analyses were performed in R version 4.4.1 (R Foundation for Statistical Computing, Vienna, Austria) using Posit Workbench version 2023.09.0+463.pro11 (Posit Software, Boston, MA, USA.

## 3. Results

### 3.1. Patient Population and Enrolment

**Figure 4:**
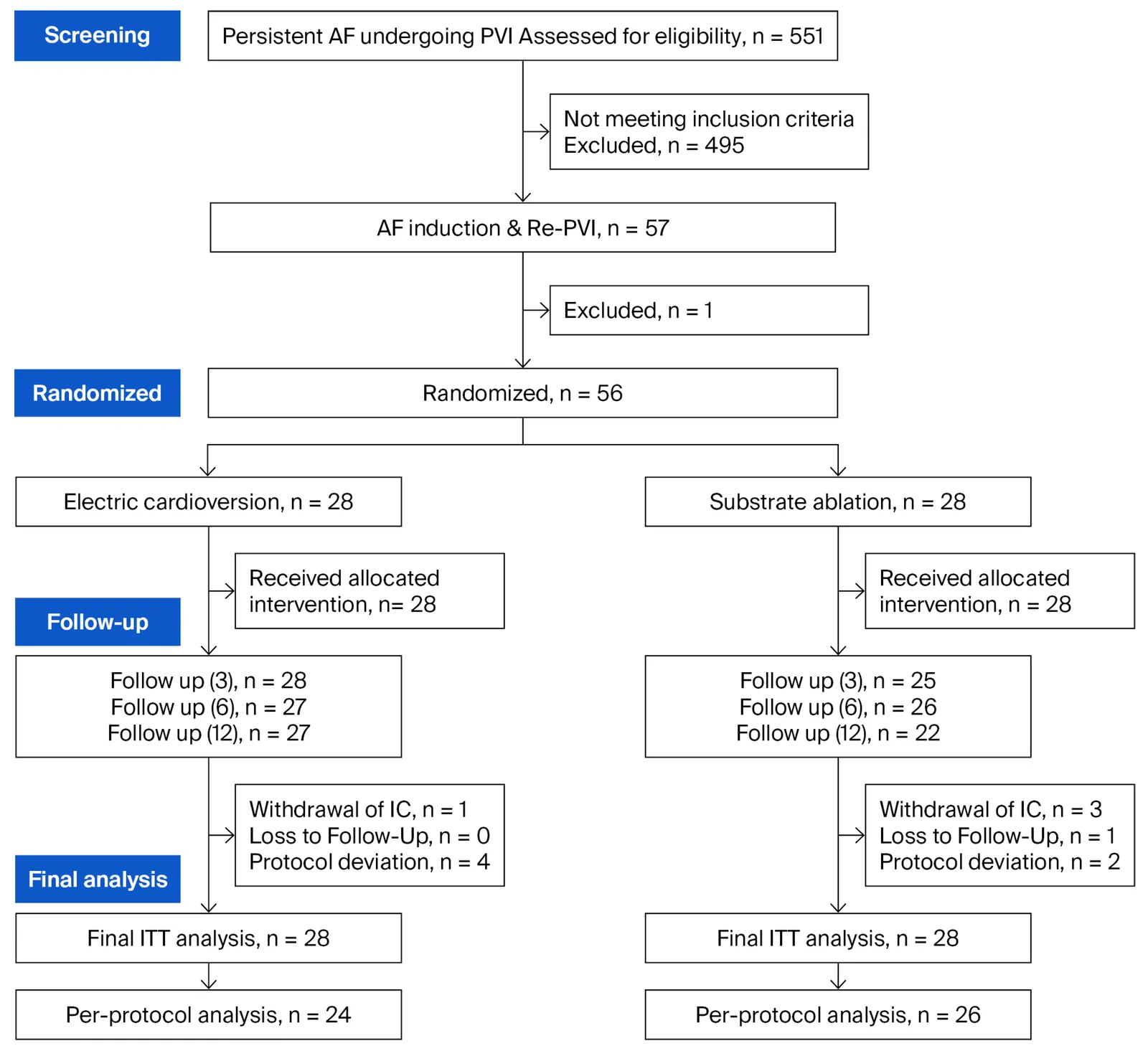
CONSORT flow diagram.

Patient enrolment began in August 2021 and ended prematurely in November 2023 after the funder discontinued financial support because of slow recruitment. In two enrolling centers, a total of 56 patients were randomized (mean age 68 ± 10 years, 32% women, mean LVEF 53 ± 8%, mean LA diameter 44.5 ± 6.2 mm), with 28 patients allocated to group A (Re-PVI only) and 28 patients to group B (Re-PVI + Substrate Ablation); see Figure 4.

Baseline characteristics by randomized group are shown in Table 1.

Numerical differences were observed for BNP, AFEQT score, diabetes, cardiomyopathy, and prior ablation modality. No formal hypothesis tests of baseline characteristics were performed.

In deviation from the protocol, six patients who were in sinus rhythm and had non-inducible AF after Re-PVI were nevertheless randomized: four to Group A and two to Group B. All six were retained in the intention-to-treat analysis and excluded from the per-protocol analysis (see Figure 4).

### 3.2. Procedural Characteristics

All patients underwent the Re-PVI procedure. According to the protocol, if AF was ongoing or inducible after completion of Re-PVI, patients were randomized during the procedure in a 1:1 ratio to either the Re-PVI only group (group A) or the Re-PVI plus substrate ablation group (group B)). Procedural characteristics are shown in Table 2.

### 3.3. Primary Endpoint

**Figure 5:**
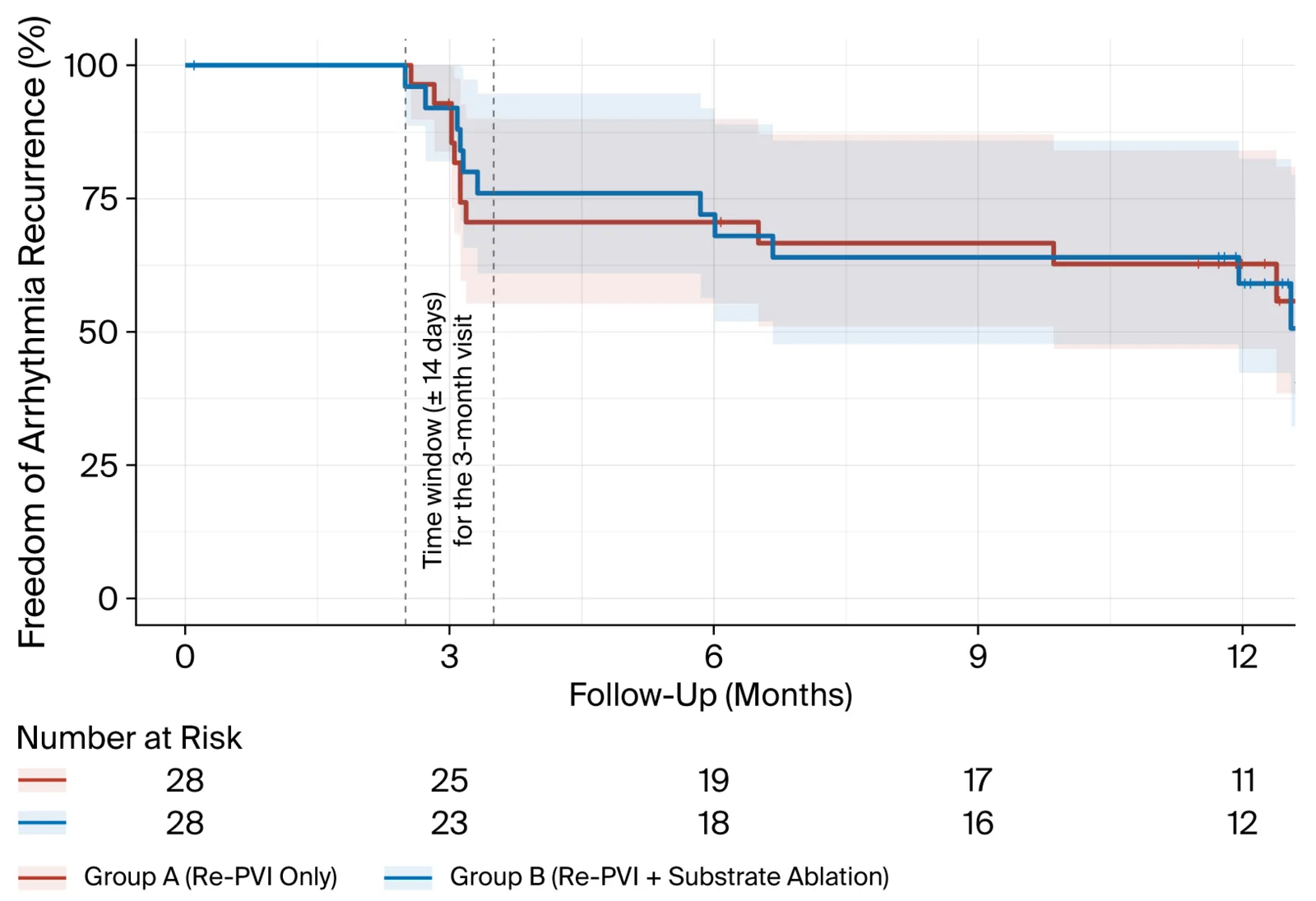
The Kaplan–Meier estimates (95% confidence intervals) for freedom from any atrial arrhythmia (>30 s). The 1-year estimates were 62.7% [46.8%, 84.1%] in group A (10 events) and 59.1% [42.3%, 82.4%] in group B (10 events), with no difference between the groups (*p* = 0.85). The confidence intervals for Group A and Group B are shown in red and blue, respectively. Areas where the confidence intervals overlap appear grey.

The median follow-up was 12.42 months [95% CI: 12.12–12.64]. The primary endpoint, freedom from atrial arrhythmia (>30 s) between 3 and 12 months post-ablation, was observed at a similar rate in both groups. In the intention-to-treat analysis, substrate ablation did not affect the risk of atrial arrhythmia recurrence compared with standard of care (HR, 1.07; 95% CI, 0.50–2.33; *p* = 0.85). Findings from the per-protocol analysis, excluding six patients who did not fulfill the inclusion criteria, were consistent with those of the primary analysis (HR, 1.21; 95% CI, 0.52–2.81). In the intention-to-treat population, the 1-year Kaplan–Meier estimate of freedom from arrhythmia was 62.7% (95% CI: 46.8–84.1) in Group A (Re-PVI only) and 59.1% (95% CI: 42.3–82.4) in Group B (Re-PVI + Substrate Ablation), as shown in Table 3 and Figure 5.

### 3.4. Secondary Endpoints and Clinical Outcomes

No differences were observed for secondary arrhythmia-related endpoints, including freedom from atrial fibrillation and freedom from atrial tachycardia.

In within-group analyses, LVEF increased from baseline in Group A at both 6 months (estimated change, 4.23 percentage points; 95% CI, 1.56 to 6.91; *p* = 0.002) and 12 months (3.55 percentage points; 95% CI, 0.84 to 6.27; *p* = 0.011). In contrast, no within-group change in LVEF was observed in Group B at either follow-up time point.

LA diameter decreased from baseline in Group B at 6 months (estimated change, −3.70 mm; 95% CI, −7.06 to −0.35; *p* = 0.031), whereas no reduction was evident at 12 months and in Group A at either visit.

BNP concentrations were lower than baseline in both groups at 6 months (Group A: ratio, 0.368; 95% CI, 0.215 to 0.629; *p* < 0.001; Group B: ratio, 0.347; 95% CI, 0.193 to 0.624; *p* < 0.001), corresponding to an estimated 63.2% and 65.3% reduction from baseline. Compared to baseline, the reduction at 12 months was 59.3% in Group A (ratio, 0.407; 95% CI, 0.235 to 0.705; *p* = 0.002) and 39.1% in Group B (ratio, 0.609; 95% CI, 0.335 to 1.109; *p* = 0.104).

In the baseline-adjusted analysis, the estimated difference in LVEF between Group B and Group A was −1.833 percentage points at 6 months (95% CI, −5.099 to 1.433; *p* = 0.268) and −4.009 percentage points at 12 months (95% CI, −7.307 to −0.710; *p* = 0.018). These secondary-outcome analyses were exploratory and were not adjusted for multiple comparisons. Key 12-month outcomes are summarized in Table 3. Paired baseline and 12-month measurements were available for 43 patients for AFEQT, 50 for LVEF, 46 for left atrial diameter, and 40 for BNP.

No deaths or major procedure-related complications occurred. Eleven serious adverse events (SAEs) were reported in 20% of patients, similarly distributed between Group A (n = 5) and Group B (n = 6; *p* = 0.85). These included one cardiac arrest, one air embolism, one acute coronary syndrome, one MI, one symptomatic ventricular fibrillation, and two typical atrial flutters. All SAEs resolved without long-term sequelae.

All 28 patients assigned to Group B underwent bilateral atrial mapping. Details are provided in the Supplementary Materials Section S1. There was no correlation between pulmonary vein reconnection and the mapping parameters.

Additionally, pulmonary vein reconnection did not have an effect on the primary endpoint (HR 0.927, CI [0.275, 3.120]).

## 4. Discussion

In this prospective, randomized trial of patients with persistent AF undergoing a second ablation procedure, the addition of targeted substrate ablation guided by CFA and DOA mapping to Re-PVI showed no evidence of improvement in freedom from atrial arrhythmia at 12 months. However, the trial was terminated early after enrollment of only 56 patients, substantially fewer than the planned sample size of approximately 188 to 200 patients, leaving the study markedly underpowered for its primary endpoint. Therefore, the neutral result should be interpreted cautiously and should not be presented as evidence of lack of efficacy of the substrate-guided strategy. Within these limitations, the study provides several important insights into the management and underlying mechanisms of persistent AF.

One of the key findings of our study is the disconnect between PV status and clinical outcomes. We observed a very high rate of PV reconnection (86%), confirming that it is a common finding in patients with AF recurrence within the era represented by our patient cohort [20,21,22]. However, the presence of PV reconnection did not predict arrhythmia recurrence after the repeat procedure. Furthermore, there was no correlation between PV reconnection and the electrical properties of the extra-pulmonary vein atrial substrate. Taken together, these findings are compatible with a contribution of the extra-pulmonary vein atrial substrate, but do not establish its relative importance compared with PV-related mechanisms [23,24].

The neutral primary outcome should be interpreted in the context of both the limited statistical power of this prematurely terminated trial and the heterogeneous evidence for adjunctive substrate modification in persistent AF. In STAR AF II, adding ablation of complex fractionated electrograms or linear lesions to PVI during an initial ablation did not improve freedom from recurrent AF compared with PVI alone [2]. Unlike STAR AF II, our study evaluated a repeat procedure and used CFA and DOA mapping to select additional ablation targets rather than applying the same substrate-modification strategies.

Results from other randomized trials also differ by the substrate targeted. ERASE-AF reported fewer atrial arrhythmia recurrences with individualized low-voltage myocardium ablation added to PVI, whereas DECAAF II found no significant reduction in recurrence with MRI-guided fibrosis ablation and reported more safety events in the adjunctive-ablation group. These approaches target low-voltage tissue and MRI-defined fibrosis, respectively, and are therefore not directly equivalent to CFA- and DOA-guided ablation. Moreover, both trials evaluated patients undergoing an initial ablation rather than the repeat-ablation population studied here [16,25].

Several mechanisms may account for the observed findings, although they cannot be established by this trial. Re-isolation of reconnected pulmonary veins may have addressed an important source of arrhythmia recurrence in both treatment groups. At the same time, the lack of an observed association between PV reconnection and subsequent recurrence does not establish that PV triggers are unimportant: reconnection was highly prevalent, and the small sample size limited our ability to assess its prognostic value.

Despite this refined strategy, the addition of CFA- and DOA-guided ablation did not result in a statistically significant improvement in the primary endpoint compared with Re-PVI alone. Given the study’s limited statistical power, this finding does not establish that the approach is ineffective. Rather, the findings remain inconclusive and may reflect insufficient power, the possibility that CFA and DOA are markers of diseased atrial tissue rather than critical drivers of the arrhythmia [26,27], limitations of the targeting strategy, or a combination of these factors. Nevertheless, the 1-year arrhythmia-free survival rates of approximately 60% in both arms are consistent with outcomes reported in meta-analyses and studies for repeat procedures in this setting [7,28].

Additionally, AFEQT scores [29,30] increased and BNP levels decreased during follow-up in the studied patients, a key indicator of myocardial wall stress and heart failure [31]. These observed changes may be clinically relevant and in line with the literature [29,30,31], but, in the absence of a non-ablation control group, they cannot be causally attributed to repeat ablation.

These findings underscore the need for larger, adequately powered trials to evaluate refined substrate ablation strategies, potentially integrating multi-modal mapping and continuous monitoring to better identify and neutralize functional drivers of the remodeled atrial substrate [16,25,32].

### Study Limitations

The primary limitation is this study’s early termination due to slow enrolment. Only 56 patients were randomized, compared with the planned sample size of 188 patients and a target enrolment of 200 patients, leaving the trial substantially underpowered for its primary endpoint. Consequently, the absence of a statistically significant between-group difference in the primary endpoint should be interpreted cautiously: given the limited statistical power, these findings neither establish that targeted substrate ablation is ineffective nor exclude a clinically meaningful benefit or harm. Additionally, while endpoint adjudication was blinded, the open-label procedure could introduce performance bias. The limited number of events and recruitment across centers precluded a reliable assessment of center- or operator-specific effects. Operator-dependent variation in mapping and target identification therefore cannot be excluded.

Relying on intermittent 48-h Holter monitoring, rather than continuous monitoring like ILRs, may have underestimated asymptomatic AF recurrence and AF burden.

Methodologically, targeting the top 10% of CFA and DOA values was a pragmatic but arbitrary threshold, which may have been insufficient to modify the critical arrhythmogenic substrate. Furthermore, the custom CFA/DOA algorithms were developed for this study and lack independent validation. This raises uncertainty about their specificity for true drivers versus markers of remodeling; future studies should externally validate these metrics.

Despite randomization, several baseline characteristics differed numerically between groups. Given the small sample and only 20 primary endpoint events, these imbalances and the limited scope for covariate adjustment should be considered when interpreting the treatment estimate.

The numerous secondary and exploratory comparisons were not adjusted for multiplicity. Consequently, isolated findings, including the observed between-group difference in LVEF at 12 months, may reflect chance and should be regarded as hypothesis-generating rather than confirmatory. The regional mapping findings are descriptive and exploratory. Because measurements from different regions within a patient are correlated and no formal within-patient regional comparison was performed, apparent regional differences should not be interpreted as evidence of anatomical differences.

Finally, a protocol deviation occurred: six patients in sinus rhythm with non-inducible AF after Re-PVI were incorrectly randomized (four in Group A and two in Group B). The direction and magnitude of any effect of their inclusion on the estimated treatment effect cannot be determined from these data.

## 5. Conclusions

In this randomized trial, which was terminated early after enrollment of 56 patients and was consequently substantially underpowered for its primary endpoint, a strategy of Re-PVI combined with targeted ablation guided by continuous fractionated activity (CFA) and dispersion of activity (DOA) did not demonstrate an improvement in freedom from atrial arrhythmia compared with Re-PVI alone. The small sample size and imprecise treatment estimate preclude a definitive conclusion about the efficacy of the adjunctive strategy. AFEQT scores increased and BNP levels decreased over follow-up in the studied patients. The high prevalence of PV reconnection, together with the absence of a clear association with subsequent arrhythmia recurrence in this small cohort, may be consistent with a contribution of the atrial substrate. These findings emphasize the continued need for adequately powered studies and more refined strategies that can more effectively identify and eliminate the true drivers of persistent atrial fibrillation.

## Figures and Tables

**Table 1:** Baseline characteristics. Continuous variables are presented as mean ± SD for approximately normally distributed data and median [IQR] for non-normally distributed data. Categorical variables are presented as n (%). The number of missing values is shown.

Parameter	Overall (n = 56)	Group A (n = 28)	Group B (n = 28)
Age, Mean ± SD	68 ± 10	67 ± 9	68 ± 10
Women, n (%)	18 (32)	8 (29)	10 (36)
BMI, mean ± SD	28.2 ± 4.2	27.4 ± 3.6	29.0 ± 4.6
Missing	1	1	0
Cardiomyopathy, n (%)	3 (5)	0 (0)	3 (11)
Arterial hypertension, n (%)	39 (70)	19 (68)	20 (71)
Coronary artery disease, n (%)	13 (23)	6 (21)	7 (25)
Diabetes mellitus, n (%)			
Type 1	1 (2)	1 (4)	0 (0)
Type 2	6 (11)	0 (0)	6 (21)
Thromboembolic events, n (%)	4 (7)	3 (11)	1 (4)
CHA2DS2-VASc-Score, median [IQR]	3.0 [1.0–3.0]	2.5 [1.0–3.0]	3.00 [1.75–4.00]
Type of previous PVI, n (%)			
conventional RF	34 (65)	19 (76)	15 (56)
RF-HSPD	2 (4)	0 (0)	2 (7)
Cryo	15 (29)	5 (20)	10 (37)
Missing	5	4	1
EHRA Score, n (%)			
I	3 (6)	1 (4)	2 (8)
II	40 (77)	22 (81)	18 (72)
III	9 (17)	4 (15)	5 (20)
Missing	4	1	3
Antiarrhythmic medication (baseline) n (%)	5 (9.4)	2 (7.4)	3 (12)
Missing	3	1	2
LVEF, % [IQR]	55 [49–59]	55 [48–58]	54 [50–60]
LA diameter, mm ± SD	44.5 ± 6.2	43.8 ± 4.7	45.3 ± 7.5
Missing	1	0	1

**Table 2:** Procedural characteristics. Continuous variables are presented as median [IQR] and categorical variables as n (%). Successful PV isolation is shown as the number of patients with PV reconnections (N). *p*-values were calculated using the Wilcoxon rank-sum test for continuous variables and the chi-square test or Fisher’s exact test, as appropriate, for categorical variables.

Characteristic	Overall (n = 56)	Group A (Re-PVI Only, n = 28)	Group B (Re-PVI + Substrate, n = 28)	*p*-Value
Procedure Duration, min [IQR]	123 [87–164]	87 [71–110]	161 [140–218]	<0.001
Fluoroscopy Time (LA), min [IQR]	13 [6–31]	6 [3–8]	32 [19–55]	<0.001
PV Reconnection Rate, n (%)	48 (86)	24 (86)	24 (86)	>0.99
Successful PV Re-isolation, n (%)	44 (92)	22 (92)	22 (92)	>0.99
No need to reisolate	8	4	4	
Complications, n (%)	1 (1.8 *)	0 (0)	1 (3.6 *)	>0.99

* Air embolism of right coronary artery (resolved).

**Table 3:** Secondary endpoints (AFEQT, LVEF, LA diameter, BNP) at the 6- and 12-month visits, compared between groups. Values are estimated marginal means (EMM) and 95% confidence intervals derived from baseline-adjusted mixed models including treatment group, visit, treatment-by-visit interaction, and baseline value as a covariate. Between-group differences represent adjusted mean differences (AMD) and 95% confidence intervals. The BNP variable was log-transformed prior to analysis. EMM and AMD effects were derived on the log scale and back-transformed to the original scale for presentation. * So AMD values represent geometric mean ratios. N is the number of observations actually used in the analysis.

Outcome Parameter	Visit (Months)	Estimated Marginal Means (95% CI)		Adj. Mean Differences (* Ratios)	*p*-Value
AFEQT Total Score, n = 43	12	74.9 (66.1, 83.6	76.4 (66.5, 86.3)	1.5 (−12.2, 15.3)	0.822
LVEF (%), n = 50	6	57.1 (54.8, 59.3)	55.2 (52.9, 57.6)	−1.8 (−5.1, 1.4)	0.268
LVEF (%), n = 50	12	56.3 (54.1, 58.6)	52.3 (50.0, 54.7)	−4.0 (−7.3, −0.7)	0.018
LA Diameter (mm), n = 46	6	41.8 (38.8, 44.8)	41.0 (37.9, 44.2)	−0.8 (−5.2, 3.6)	0.718
LA Diameter (mm), n = 46	12	43.4 (40.2, 46.5)	43.4 (40.1, 46.6)	0.0 (−4.5, 4.5)	0.996
BNP (pg/mL), n = 40	6	232 (136, 396)	234 (131, 418)	1.01 (0.47, 2.16) *	0.986
BNP (pg/mL), n = 40	12	263 (151, 456)	464 (257, 838)	1.77 (0.80, 3.89) *	0.154

## Data Availability

The data presented in this study are available on request from the corresponding author. The data are not publicly available due to data protection restrictions.

## Supplementary Materials

The following supporting information can be downloaded at: https://www.mdpi.com/article/10.3390/jcm15208010/s1, Section S1: Substrate mapping parameters (group B only); Section S2: Safety and Adverse Events: Table S1: Anticoagulation, antiarrhythmics; Table S2: Serious adverse events.
