# Supplementary material for: Repeat Ablation for Persistent Atrial Fibrillation: CFA/DOA-Guided Substrate Ablation Versus Re-PVI Alone in the Prematurely Terminated Randomized AF-CAM Trial
Source: J Clin Med. 2026 Oct 15;15(20):8010. doi: 10.3390/jcm15208010 (PMC13705792; doi:10.3390/jcm15208010)
Supplement: Supplementary file 1 [file jcm-15-08010-s001.zip › jcm-4550034-supplementary.pdf]

AF-CAM study: Supplementary Materials

S1: Substrate mapping parameters (group B only)

Regional values are presented descriptively; apparent differences between regions were not formally tested.

Regional electrical parameters in the left atrium (LA) showed the highest mean voltage amplitude (0.759 ± 0.475 mV) and DOA (0.388 ± 0.031) in the anterior region, with the lowest voltage (0.538 ± 0.373 mV) in the inferior region and DOA (0.351 ± 0.034) in the posterior region. CFA was highest (0.078 ± 0.019) in the inferior region and lowest (0.066 ± 0.025) in the lateral region, while the mean AF cycle length ranged from 197 ± 19 ms (septal) to 186 ± 21 ms (roof). In the right atrium (RA), the lateral region exhibited the highest voltage (1.322 ± 0.558 mV), and the septal region the lowest (0.838 ± 0.349 mV), with DOA peaking at 0.352 ± 0.030 (septal) and lowest at 0.338 ± 0.032 (posterior). CFA was highest (0.060 ± 0.018) in the anterior region and lowest (0.051 ± 0.012) in the lateral region, with AF cycle length ranging from 203 ± 19 ms (septal) to 193 ± 19 ms (anterior).

S2: Safety and Adverse Events

No deaths or major procedure-related complications occurred. Eleven serious adverse events (SAEs) were reported in 20% of patients, similarly distributed between Group A (n=5) and Group B (n=6; p=0.85). These included one cardiac arrest, one air embolism, one acute coronary syndrome, one MI, one symptomatic ventricular fibrillation, and two typical atrial flutters. All SAEs resolved without long-term sequelae.

Table S1: **Anticoagulation, antiarrhythmics.**

**Characteristic**	**N**	**Overall**, N = 56	**Group A (Re-PVI Only)**, N = 28	**Group B (Re-PVI + Substrate Ablation)**, N = 28
Anticoagulation (baseline), % (n)	55	100 (55)	100 (27)	100 (28)
Anticoagulation (discharge), % (n)	56	100 (56)	100 (28)	100 (28)
Anticoagulation (3 months), % (n)	53	98 (52)	96 (27)	100 (25)
Anticoagulation (6 months), % (n)	52	90 (47)	89 (24)	92 (23)
Anticoagulation (12 months), % (n)	51	92 (47)	89 (24)	96 (23)
Antiarrhythmics (baseline), % (n)	53	9.4 (5)	7.4 (2)	12 (3)
Antiarrhythmics (discharge), % (n)	56	20 (11)	21 (6)	18 (5)
Antiarrhythmics (3 months), % (n)	53	9.4 (5)	14 (4)	4.0 (1)
Antiarrhythmics (6 months), % (n)	52	1.9 (1)	3.7 (1)	0 (0)
Antiarrhythmics (12 months), % (n)	51	3.9 (2)	3.7 (1)	4.2 (1)

Categorical data are presented with percentages and frequencies.

Table S2: Serious adverse events.

**Characteristic**	**N**	**Group A: control group**, N = 15	**Group B: substrate ablation**, N = 16
SAE Criteria, % (n)	31		
death		0 (0)	0 (0)
life threatening		6.7 (1)	13 (2)
hospital admission/ prolonged stay		93 (14)	88 (14)
disability		0 (0)	0 (0)
congenital disorder		0 (0)	0 (0)
other serious medical condition		0 (0)	0 (0)
SAE resolved, % (n)	28	100 (14)	100 (14)
Unknown		1	2
Study-related complications, % (n)	31		
related		67 (10)	63 (10)
possible		0 (0)	0 (0)
unlikely		6.7 (1)	6.3 (1)
not related		27 (4)	31 (5)
SAE causality to procedure, % (n)	31		
related		0 (0)	0 (0)
possible		67 (10)	69 (11)
unlikely		6.7 (1)	6.3 (1)
not related		27 (4)	25 (4)
SAE cardiac cause, % (n)	31	80 (12)	88 (14)
SAE cardiac cause definition, % (n)	31		
ACS		6.7 (1)	0 (0)
arrhythmia		73 (11)	75 (12)
heart failure		0 (0)	0 (0)
other		20 (3)	25 (4)

Categorical data are presented with percentages and frequencies.
